# Heightened ACE Activity and Unfavorable Consequences in COVID-19 Diabetic Subjects

**DOI:** 10.1155/2020/7847526

**Published:** 2020-07-16

**Authors:** Rashi Sandooja, Naga Venkata Rama Krishna Vura, Michael Morocco

**Affiliations:** ^1^Department of Internal Medicine, Cleveland Clinic Akron General, Akron, Ohio, USA; ^2^Division of Geriatrics and Palliative Care, Department of Medicine, Rhode Island Hospital, Warren Alpert Medical School of Brown University, Providence, USA; ^3^Department of Endocrinology, Cleveland Clinic Akron General, Akron, Ohio, USA

## Abstract

Coronavirus disease-19 (COVID-19) can manifest as fever, cough, and shortness of breath and is caused by severe acute respiratory syndrome coronavirus-2 (SARS-CoV-2). Occasionally, these patients may present with diabetic ketoacidosis (DKA). Patients with comorbidities such as diabetes mellitus and hypertension, as well as older age groups, are known to have a more severe disease progression and poor prognosis. In this review article, we attempt to better understand the disease process in diabetics and also discuss potential mechanisms by which diabetes may play a role in disease severity. This includes the effect of diabetes on the immune response and immune dysregulation. The role of diabetes mellitus in molecular processes including augmenting Angiotensin-Converting Enzyme 2 (ACE 2) levels is also discussed as potential mechanisms to explain the grave prognosis seen in these patients.

## 1. Introduction

The pandemic of coronavirus disease-19 (COVID-19) has been associated with substantial morbidity and mortality. The causative agent of COVID-19 has been known to be severe acute respiratory syndrome coronavirus-2 (SARS-CoV-2). It is a novel enveloped ribonucleic acid (RNA) beta-coronavirus. The disease was first reported in Wuhan, Hubei Province of China, in December 2019, after a cluster of cases with unknown causes of pneumonia that did not respond to usual treatments. By the end of January 2020, the WHO declared the disease as pandemic. As of May 1, 2020, the disease had spread to 187 countries, affecting 3.3 million individuals and having caused more than 230,000 deaths.[1].

Many risk factors have been identified to be associated with higher severity and mortality of the disease. These include hypertension, diabetes mellitus, older age, and severe obesity.

In this review article, we try to provide a better understanding of COVID-19 disease in patients with diabetes and possible molecular mechanisms in diabetics that may affect the disease severity and pathogenesis.

## 2. Epidemiology

In the U.S., 10.5% of the total population of 34.2 million individuals is known to have diabetes [2]. In addition, 68.4% of the population with diabetes also has coexisting hypertension, and 15.5% of diabetics also has severe obesity [3]. Due to the high prevalence of diabetes in the population along with the coexistence of hypertension and obesity with diabetes, it is essential to have a better understanding of the disease process in this subset of individuals. A study conducted in Hong Kong showed that the mortality rates from pneumonia in diabetics aged 75 and above were even higher than the mortality rate from cardiovascular disease and cancer in the same age group [4]. Various studies in the past have tried to look at the association between diabetes and the severity of COVID infections.

In a retrospective, multicenter cohort study of 191 COVID-19-positive patients conducted in two hospitals of Wuhan, China, 19% of the patients admitted had diabetes, with diabetes being the second most common comorbidity after hypertension. Out of the total 54 patients who died in this study, 31% of the patients had diabetes [5]. In another study conducted in China, out of a total of 1,099 patients with confirmed COVID-19 disease, 173 had severe disease. Patients with severe disease were found to have a higher prevalence of diabetes mellitus [6]. On a similar note, in Italy, more than two-thirds of the patients dying from SARS-CoV-2 were known to have diabetes, cardiovascular disease, or cancer [7].

## 3. Pathogenesis

SARS-CoV-2 is the third coronavirus member, which circulated extensively in the last three decades. Previously known highly pathogenic coronavirus outbreaks have been the Severe Acute Respiratory Distress (SARS) in 2002 and the Middle East Respiratory Syndrome (MERS) in 2012. Both these outbreaks were known to be caused by the SARS-CoV-1 and MERS-CoV, respectively.

Genetic sequencing of SARS-CoV-2 showed more than 80% similarity to the previous SARS-CoV-1 and almost 50% similarity to MERS-CoV. It is reasonable to thus assess similarities in the disease-causing process of the novel SARS-CoV-2 to its previous pathogenic counterparts at the molecular level.

SARS-CoV-2 is made up of four structural proteins, namely, the spike protein, membrane protein, nucleocapsid, and envelope protein. The cellular receptor responsible for the binding of SARS-CoV-2 is the Angiotensin-Converting Enzyme 2 (ACE 2) receptor [8]. This is in contrast to MERS-CoV where the cellular receptor was the dipeptidyl peptidase IV (DPP-IV) receptor [9]. The ACE 2 receptors on the host cells interact with the spike glycoprotein on the virus, facilitating viral entry into the host cells. ACE 2 receptors are found in the cardiovascular system, kidneys, lungs, and brain [10]. Next after viral binding to ACE 2 receptors is the role of serine proteases such as transmembrane protease serine (TMPRSS) that is involved in protein priming and cleavage of the spike protein. Proteases such as Furin release the spike fusion peptide, enabling virus entry into the endosomal pathway.

Interestingly, low pH favors the entry of the SARS-CoV-2 into the cytosol and thus viral replication [3].

## 4. Clinical Presentation

The most common presentation of COVID-19 is fever, cough, and shortness of breath. Uncommonly, patients with COVID-19 can present with diabetic ketoacidosis (DKA) as the presenting symptom. This may complicate the management of patients presenting with DKA, as excessive fluid resuscitation may potentiate acute respiratory distress syndrome (ARDS) [11]. Another study of 658 hospitalized patients with confirmed COVID-19 disease concluded that this infection causes ketosis or ketoacidosis and induced DKA in diabetic patients [12].

Theoretically, COVID-19 infection can present as an acute adrenal crisis, especially in patients with known chronic adrenal insufficiency, which emphasizes the importance of reiteration of sick day rules to patients on chronic steroids.

## 5. Diabetes and COVID-19

Various studies have tried to look at the association between diabetes and disease severity. A retrospective cohort study of 201 patients, conducted in Wuhan, looked at the factors predicting disease progression to ARDS. This study identified that patients who developed ARDS had a higher incidence of comorbidities. 19% of the patients who developed ARDS had diabetes mellitus in comparison to 5.1% of the patients who did not develop ARDS had diabetes [4].

The progression of COVID-19 in this special population can be attributed to the following factors.

### 5.1. Diabetes and Immune Response

Diabetes mellitus is a low-grade chronic inflammatory state, due to the excessive visceral adipose tissue. COVID-19 patients with diabetes mellitus are at a higher risk of an excessive uncontrolled inflammatory response and hypercoagulable state.

In a prior study by Kulcsar et al., mice, in their model, were made susceptible to MERS-CoV by expressing DPP-IV receptors, and type 2 diabetes mellitus was induced by administering a high-fat diet. It was seen in this study that diabetic mice had prolonged, severe disease with delayed recovery [13]. This further concluded that diabetes is associated with dysregulated immune response leading to severe and prolonged disease. Laboratory abnormalities observed in COVID-19 patients such as a low cluster of differentiation (CD) CD 4+, CD 8+ counts, and elevated cytokine levels prove that innate immunity against SARS-CoV-2 is compromised in diabetes. This, then, leads to decreased mobilization of polymorphonuclear leukocytes (PMN), decreased chemotaxis, decreased phagocytic activity, and inhibition of tumor necrosis factor (TNF) alpha activity [14].

Previous studies have shown COVID-19 patients presenting with different biochemical abnormalities. In a study of 1,099 patients conducted in COVID-19-positive patients in China, admission labs were significant for lymphopenia in 83.2% of the patients, thrombocytopenia in 36.2% of the patients, and leukopenia in 33.7% of the patients [6]. Earlier studies have also shown that SARS-CoV-2, like its predecessor SARS-CoV-1, used the same ACE 2 receptors and, in this way, can infect immune cells such as T-cells, macrophages, and monocytes. This may contribute to lymphopenia that is commonly seen in COVID-19 patients.

Most of the patients in the abovementioned study also had elevated levels of C-reactive protein (CRP); less common were elevations in alanine transferase (ALT), aspartate transferase (AST), creatine kinase (CK), and d-dimer. It was also seen that patients with severe disease had more prominent laboratory abnormalities as compared to patients with nonsevere disease [6]. These biochemical markers have since then been used to correlate with disease progression and severity.

In another retrospective study of 174 patients in Wuhan, that looked at diabetes as a risk factor in progression and prognosis of COVID-19, it was seen that levels of inflammatory markers such as CRP, erythrocyte sedimentation rate (ESR), d-dimer, and interleukin-6 (IL-6) were significantly higher in diabetics compared to nondiabetic patients [15]. These patients also had higher lactate dehydrogenase (LDH), alpha-hydroxybutyrate dehydrogenase (HBDH), ALT, and gamma-glutamyl transferase (GGT) compared to their nondiabetic counterparts, indicating further damage to the myocardium, kidneys, and liver. The study also further showed that lymphocyte count, red blood cell (RBC) count, and hemoglobin (Hb) were all significantly lower in diabetics. The authors of the study also further note that diabetic patients had lower protein, prealbumin, albumin, and Hb levels, signifying undernourishment, thus contributing to poor prognosis of such patients.

### 5.2. Diabetes Affecting Cellular Receptors

As mentioned previously, the ACE 2 receptors have been identified as the viral-binding receptors for the novel SARS-CoV-2. Of note, ACE is responsible for the conversion of angiotensin I to angiotensin II, whereas ACE 2 metabolizes angiotensin II to angiotensin 1–7. Angiotensin II is responsible for vasoconstriction and cell proliferation.

In a previous study by Roca-Ho et al., looking at ACE and ACE 2 expression in a nonobese diabetic mouse model, it was seen that diabetes upregulated ACE levels mainly in the serum, lung, heart, and liver and ACE 2 levels in the serum, liver, and pancreas [16]. Another study by Wyoscki et al., studying ACE and ACE 2 activity in diabetic mice, concluded that, in diabetes, ACE 2 levels are increased at the posttranscriptional level [17]. It is this increase in the pulmonary ACE/ACE 2 ratio that is observed in ARDS. Viral binding of SARS-CoV-1 with the ACE 2 receptors causes unopposed angiotensin II activity, which may contribute to acute lung injury.

Medications may also play a role in the severity of disease associated with diabetes. Home medications of diabetics often include hypoglycemic agents such as insulin and glucagon-like peptide-1 (GLP-1) analogues. These patients also frequently have ACE inhibitors as part of their treatment list.

**Figure 1:**
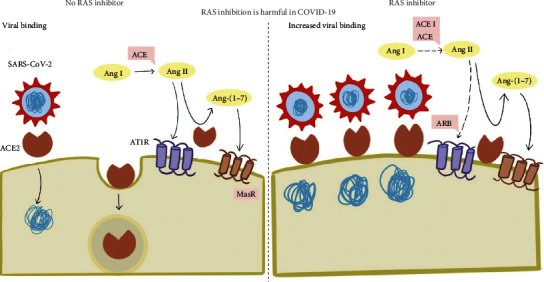
Schematic diagram representing the possible effects of renin-angiotensin-adosterone-system (RAAS) inhibition on ACE expression. SARS-CoV-2 enters the host cell via the ACE 2 receptors causing downstream effects. Prior use of RAAS inhibitors is associated with the upregulation of the ACE 2, increasing viral entry into the host cell. Moreover, following viral entry, ACE 2 gets downregulated. Hypothetically, this leads to reduced ACE 2 availability for clearance of angiotensin II, resulting in acute lung injury.

Roca-Ho et al. also noted that the administration of insulin was associated with restoration of the ACE and ACE 2 levels in the serum, which may indicate a protective effect of insulin [16]. Other animal studies looking at GLP-1 analogues (liraglutide), ACE inhibitors, and statins showed upregulation of ACE 2 expression associated with these drugs [18–20]. Theoretically, we propose that this augmented ACE 2 expression favors increased cellular binding of SARS-CoV-2 to the host cells (see Figure 1). This may indicate higher susceptibility for an inflammatory storm in diabetic patients affected with SARS-CoV-2 leading to more severe disease.

Moreover, in a study looking at viral clearance, conducted in China, of 106 hospitalized COVID-19-positive patients, the median time for a negative SARS-CoV-2 RNA was 15 days. It was concluded in this study that factors affecting ACE 2 expression, such as diabetes and patients on ACE inhibitors, were associated with delayed viral clearance [21]. This prolonged viral clearance may also be a contributing factor to poor prognosis seen with diabetes and COVID-19 infection.

As of April 2020, due to lack of conclusive evidence with respect to risk vs. benefits, no guidelines exist for patients testing positive for COVID-19 who may be on one or more of the abovementioned drugs such as ACE inhibitors, statins, or GLP-1 analogues.

DPP-IV is another important receptor that may play a role in the pathogenesis of COVID-19. DPP-IV is a transmembrane glycoprotein that plays a major role in glucose metabolism. It acts by degrading incretins such as GLP-1, which, in turn, leads to reduced insulin secretion. It also increases inflammation in type 2 diabetes. DPP-IV has been known to be the receptor for viral binding for MERS-CoV. The odds ratio of developing severe complications of MERS-CoV ranged between 2.47 and 7.24 in diabetics [14]. Studies using mouse models with induced diabetes and expressing DPP-IV showed to have a prolonged phase of severe disease with MERS-CoV. However, the effect of DPP-IV inhibitors, if any, on inflammation and the risk of infection with SARS-CoV-2 is not yet clearly understood.

### 5.3. Effects of Hyperglycemia and Hypoglycemia

Diabetes mellitus affects the innate immune response by causing dysregulation of the immune system. In addition, uncontrolled blood sugars levels, often seen in diabetics, are associated with adverse outcomes. Even short-term hyperglycemia can transiently stun the innate immune system and potentially explain poor outcomes, especially in critically ill patients [22]. In a retrospective cohort study of 201 patients conducted in Wuhan, China, the authors noted that blood glucose was one of the risk factors that were associated with the development of ARDS in COVID-19 patients [4].

Furthermore, a previous retrospective analysis looking at the relationship between fasting blood glucose levels with death, in patients with SARS, showed that hyperglycemia was an independent predictor for death in SARS. The authors concluded that metabolic control may lead to a better prognosis of SARS patients. Yang et al., in their retrospective study, observed that a significant percentage of insulin-requiring diabetic patients had to increase their insulin dose after admission. Similarly, patients on oral medications had to be transitioned to insulin for better glycemic control after hospitalization. This was the basis of the conclusion that SARS-CoV-2 causes glucose dysregulation and poor prognosis in diabetics [23].

Just like hyperglycemia, hypoglycemia has been associated with worse outcomes in hospitalized patients. No studies looking at the effect of hypoglycemia on outcomes for COVID-19 patients were found. However, a retrospective cohort study looking at the effect of hypoglycemia (defined by the authors as blood glucose less than 70 mg/dl) on patients admitted with community-acquired pneumonia concluded that hypoglycemia was independently associated with higher 30-day mortality for such patients. This gives rise to a fair assumption that fairly strict glycemic control is an important component of treatment that should not be missed in patients admitted for ARDS secondary to SARS-CoV-2. On these grounds, we propose strict blood glucose control in patients admitted with COVID-19, especially those who are critically ill. Further studies are warranted to look at the exact relationship between blood glucose levels and mortality in these patients.

## 6. Conclusions

In conclusion, the presence of diabetes can be associated with a severe course of SARS-CoV-2 due to the dysregulation of innate immunity. Increased expression of ACE 2 is an important factor at the molecular level that may explain the reason for adverse outcomes in these patients. This raises questions regarding patients on drugs such as GLP-1 analogues or ACE inhibitors that are known to augment the expression of ACE 2 and might contribute to disease severity in these patients.

In addition, DPP-IV receptors as potential targets in COVID-19 patients to reduce disease severity further opens up opportunities for research.

Finally, the pandemic of COVID-19 begets many other challenges faced by diabetics, which are unrelated to the disease severity and prognosis. Postponement or delayed clinic visits lead to interrupted healthcare delivery. Access to basic medical supplies and insulin became a challenge, especially for at-risk populations. The transition to telehealth visits by most of the outpatient clinics is an ingenious solution to this challenge faced worldwide. However, the rush to virtual telehealth visits presented further challenges, such as the inability to download glucose sensor data or interrogate an insulin pump via the available telehealth interfaces.

This unprecedented pandemic also raises many questions for patients with diabetes. As mentioned before, diabetics admitted to the hospital with SARS-CoV-2 saw an increase in the insulin requirements after admission. In our search of the literature, we were unable to find any studies describing the association between the duration of diabetes and the effect of glycemic control prior to admission on the disease severity and progression. The role of stricter glycemic control on an outpatient basis and stricter HbA1c goals on the prognosis is unknown and should be further studied.

Also, it is important to understand the effect of type 1 vs. type 2 diabetes mellitus on the course of the disease. As the pathology of both is substantially different, it is essential to further study this difference and its effects on COVID-19. This may substantially change the treatment course of the patients who are admitted with SARS-CoV-2 infection.
